# The Relationship Between Lockdowns and Video Game Playtime: Multilevel Time-Series Analysis Using Massive-Scale Data Telemetry

**DOI:** 10.2196/40190

**Published:** 2023-11-08

**Authors:** David Zendle, Catherine Flick, Darel Halgarth, Nick Ballou, Joe Cutting, Anders Drachen

**Affiliations:** 1 Department of Computer Science University of York York United Kingdom; 2 School of Computer Science and Informatics De Montfort University Leicester United Kingdom; 3 School of Electronic Engineering and Computer Science Queen Mary University of London London United Kingdom; 4 Faculty of Engineering University of Southern Denmark Odense United Kingdom

**Keywords:** COVID-19, lockdown policy, disordered gaming, big data, playtime, policy, lockdown, public health, side effects, pandemic, video games, playing, gaming, time, disordered

## Abstract

**Background:**

COVID-19 led governments worldwide to enact a variety of containment and closure policies. Substantial attention has been directed toward the idea that these public health measures may have unanticipated negative side effects. One proposed effect relates to video games. There is a nascent evidence base suggesting that individuals played video games for longer and in a more disordered manner during lockdowns and school closures specifically. These increases are commonly framed as a potential health concern in relation to disordered gaming. However, the evidence base regarding changes in gaming during the COVID-19 pandemic is based on self-report and, thus, is susceptible to bias. Therefore, it is unclear what the true consequences of lockdowns were for gaming behavior worldwide.

**Objective:**

The primary objective of this study was to estimate whether any specific lockdown policy led to meaningful increases in the amount of time individuals spent playing video games.

**Methods:**

Rather than relying on self-report, we used >251 billion hours of raw gameplay telemetry data from 184 separate countries to assess the behavioral correlates of COVID-19–related policy decisions. A multilevel model estimated the impact of varying enforcement levels of 8 containment and closure policies on the amount of time that individual users spent in-game. Similar models estimated the impact of policy on overall playtime and the number of users within a country.

**Results:**

No lockdown policy can explain substantial variance in playtime per gamer. School closures were uniquely associated with meaningful increases in total playtime within a country (*r*^2^=0.048). However, this was associated with increases in the number of unique individuals playing games (*r*^2^=0.057) rather than increases in playtime per gamer (*r*^2^<0.001).

**Conclusions:**

Previous work using self-report data has suggested that important increases in heavy gaming may occur during pandemics because of containment and closure (“lockdown”) procedures. This study contrasts with the previous evidence base and finds no evidence of such a relationship. It suggests that significant further work is needed before increases in disordered or heavy gaming are considered when planning public health policies for pandemic preparedness.

## Introduction

### Background

The emergence of COVID-19 led governments worldwide to enact a variety of policy decisions in an attempt to both slow the pandemic’s spread and mitigate its impact. These measures ranged from the requirement that masks be worn in specific areas to policies related to vaccination and testing, as well as fiscal measures and debt relief policies [1-4]. A key theme within policy-making concerns what are referred to as “containment and closure policies” or “lockdown policies.” These refer to governmental interventions that attempt to control the spread of SARS-CoV-2 through activity-limiting interventions that include stay-at-home orders, restrictions on gatherings, and other societal restrictions such as the closure of schools [5].

The primary aim of containment and closure policies has been to minimize the transmission of COVID-19 and, hence, its impact on population health. Substantial epidemiological attention has been paid to assessing their effectiveness in this regard [6-9]. However, substantial attention has also been paid to the idea that these policies may have important unintended or unanticipated social and behavioral consequences. Particular attention has been paid to the idea that the behaviors engendered by lockdowns may lead to significant detriment to human health and well-being, with some even suggesting that such impacts may outweigh the benefits of lockdowns [10]. Candidate effects are diverse and include the potential impact of containment and closure policies on screen time, education, alcohol consumption, diurnal rhythms, eating habits, and physical activity [11-16].

### Heavy Gaming and Lockdowns

One particularly widely discussed potential effect concerns video gaming. The primary issue concerns the amount of time that individuals spend playing video games. The idea that excessive levels of playtime may have negative consequences for individuals is common in the literature, with several studies suggesting a relationship between COVID-19 policy-making and increases in disordered or addictionlike gaming [17-21]. Particular attention has been paid to the impact of school closures in this context. Some have suggested that the confinement of young people to their homes may lead to increases in the volume of playtime among this group and that these increases may be so extreme that playtime becomes problematic [18,19]. What constitutes a problematic volume of personal playtime is not well understood in the literature [22]. Increases in playtime may not always be considered harmful, and what constitutes excessive or problematic gaming will change depending on the person involved.

The broader literature also deals with alternative changes that may have occurred to video gaming during the pandemic. On the one hand, some researchers have suggested that work-from-home mandates and other similar restrictions may lead to increased free time and decreased options for nongaming leisure pursuits, with consequent increases in playtime within a territory [17,23,24]. However, on the other hand, some academics have proposed that the impact of containment and closure policies may be driven by social needs—as policies recommend or require restrictions on social gatherings, individuals may turn to multiplayer video games as a social medium through which to mitigate potential loneliness [25-27].

Evidence regarding any of the behavioral impacts outlined previously is sparse. The analysis of large-scale public data collected from the gaming platform *Steam* has suggested that the first year of the pandemic saw an overall upturn worldwide in terms of the number of concurrent gaming sessions taking place on desktop games [28]. However, empirical studies that aim to assess the impact of policy on video game play have typically focused on samples drawn from a single country measured at 1 or 2 time points during the pandemic using self-report measures [17,18,20,29,30]. Therefore, they lack the ability to generate knowledge regarding the relative impact of the different containment and closure policies that were implemented worldwide during the COVID-19 pandemic. Furthermore, these studies are based almost universally based on self-reports of technology use rather than its measurement via objective logs, which may lead to measurement error [31]. Therefore, the overall impacts of containment and closure policies on playtime are unclear.

### Video Games and Well-Being

Although the nature of changes in playtime may be unclear, academic opinion is generally united in the belief that changes in video game play are societally important. However, as outlined previously, opinions are divided in terms of the valence of this importance. Some prior work has argued that increased levels of gaming during the pandemic may lead to increased levels of disordered gaming and, hence, lower levels of well-being [18,20,29,32]. However, other researchers have suggested that increased levels of gaming may perform a socially beneficial function as a means to socialize and deal with pandemic-related stress [30,33-35]. Indeed, some sources have even put forward the idea that commercial video games may be used as an alternative solution to traditional mental health treatment during COVID-19 containment and closure restrictions [26]. Under this approach to gaming impact, alarmist attitudes regarding the interconnection between increased gaming and disordered gaming may be detrimental to overall public health [36].

Ultimately, the emerging academic literature on the impact of policy on video game play lacks the ability to accurately estimate the differential impact of specific policy decisions on playtime worldwide because of a lack of global behavioral data regarding playtime. Overall, containment and closure policies may meaningfully be divided into 8 separate categories: school closures, workplace closures, the cancellation of public events, restrictions on gatherings, closure of public transport, stay-at-home requirements, restrictions on internal movement, and international travel controls [5]. To understand the impact of each of these policy categories, research must assess their comparative and combined global impact using large-scale and globally distributed data on playtime.

### This Research

The literature on how gaming may have affected human flourishing during the pandemic rests on behavioral foundations—the impacts on well-being proposed in the literature require substantial changes to first occur in terms of the volume of video game play per individual. If policy decisions are able to explain substantial variance in these outcomes, then such interventions are theoretically capable of causing the psychological and health impacts proposed in the literature. However, if no such effect on behavior exists, then holistic impacts on human well-being become implausible. For example, if school closures are not associated with important increases in the amount of time that individuals spend in-game, then their ability to systemically affect the health of young gamers by encouraging unhealthily extreme levels of gaming becomes significantly less plausible.

Therefore, in this study, we assessed the evidence for changes in playtime during the enforcement of a variety of lockdown policies worldwide. More formally, we investigated whether the different geographical scopes and enforcement levels of 8 separate containment and closure policies (including the closure of schools) were able to explain variance in playtime per user within a territory. We also measured the impact of policy on both total playtime within a territory and the number of players within that territory.

For this study, we used an unprecedentedly large data set provided by Unity Technologies. Unity Technologies are the makers of one of the world’s most popular game engines—the software that is used to develop and build games themselves [37]. Their data for the period under analysis cover 251.88 billion hours of playtime recorded in 184 countries worldwide and across tens of thousands of separate games. This represents all playtime in desktop and mobile games that implement Unity Analytics. Thus, although it does not represent all the playtime in all the games worldwide and does not include any console games, it is orders of magnitude larger and more geographically diverse than any playtime data ever analyzed before this point [38-40]. These data are able to measure the sum total of hours spent in all games that implement Unity Analytics worldwide. They may be decomposed by territory and date.

We combined this with data drawn from the Oxford COVID-19 Government Response Tracker (OxCGRT) database, a data set that measures day-by-day changes to the enforcement level and geographical scope of 8 containment and closure policy categories within the 184 countries in the Unity data [5]. By doing so, we were able to estimate the relative changes in playtime that were associated with the tightening or loosening of various containment and closure policies during the first 2 years of the COVID-19 pandemic.

To investigate the impact of specific policy decisions on playtime, we fitted a series of 3 separate multilevel models to data on playtime from the 184 countries in our data set. Our first model estimated the ability of policy decisions to explain the variance in the total amount of playtime occurring within a country; our second and third models decomposed this relationship by estimating the ability of policy decisions to explain the variance in the number of daily unique users within a country and the amount of playtime per user within that country.

Apart from their outcome variables (total playtime, number of users, and playtime per user), our models were otherwise identical—each contained fixed effects that allowed for modeling of the impact of differing enforcement levels and geographical scopes of the 8 containment and closure policy categories within the OxCGRT database [5]. An additional fixed effect allowed each model to account for any overall global linear trends in playtime. To account for potentially different linear trends in playtime within each individual country in our data set, each model incorporated random effects in the form of country-level slopes over time. The policy categories were school closures, workplace closures, the cancellation of public events, restrictions on gatherings, closure of public transport, stay-at-home requirements, restrictions on internal movement, and international travel controls. Each model estimated the impact of these policy categories on the total amount of playtime within a territory, the daily number of users playing video games, and the average amount of time that each of these users spent in-game. These analyses accounted for both the geographical scope of a policy (targeted to a specific area or generally enforced within a territory) and the enforcement severity of that policy (on an ordinal scale ranging from 1 to 4). More information on the modeling approach that was taken is provided in the *Methods* section.

## Methods

### Ethical Considerations

Unity Technologies collects a substantial amount of data from players using the Unity Analytics toolkit, which is an extension that needs to be enabled by developers using the Unity engine to operate. This means that not all games using the Unity engine enable analytics, but those that do prompt a short consent agreement when installing the game that explains the collection and use of these data to the player. Unity Technologies has a set of documents that explains the requirements for the collection, storage, and use of analytics data [41]. One of the uses explained in the collection agreement is for research purposes, which is the purpose under which these data have been shared with the researchers. The specific data analyzed in this study are not personally identifiable information, and the research team does not have access to personally identifiable information from Unity Analytics. The data presented in this paper are aggregated from a collection that is pseudonymized by way of a token unique to each player of each individual game—no players are traceable across games, only within a specific game. Therefore, we consider this use of data within reasonable ethical use under research ethics norms and expectations. The research has also received clearance from the University of York’s Physical Sciences Ethics Committee (approval identifier: Zendle20211021). This study consisted of the analysis of previously collected anonymized and aggregated playtime data. As such, there were no formal participants in this study.

### Data

Access to the playtime data used in this study was provided by Unity Technologies. Although it is unknown exactly how large a fraction of the video game market relies on the Unity game engine, according to Unity Technologies’ own 2021 estimates, there are approximately 5 billion downloads per month of apps (including games and nongame apps) developed using Unity, 2.8 billion monthly active end users who consume Unity-created or operated content, and Unity “remains the game engine of choice” for 61% of developers [42].

Products made using Unity’s game engine may implement Unity Analytics—a toolkit for tracking and understanding in-game engagement. Although it is theoretically possible for a Unity game to not have Unity Analytics enabled, in practice, this is thought to only occur infrequently; a substantial number of basic game functionalities cannot operate without data collection about user behavior—and Unity Analytics provides such functionality. If a user does not have an internet connection, a buffer system stores data locally on the device until the connection is re-established. Furthermore, for Unity Analytics, if a user should leave an active game running but not interact with it, a timer (20 minutes) sets in that stops collecting data for the active session. Games running in the background are not integrated into the session time.

Unity Analytics integration allows developers to understand factors such as the daily playtime associated with individual users or the frequency of a user’s in-game interactions. Although Unity does not store analytics data for Unity products that are launched on any console, it does deidentify the data that are collected by desktop and mobile games implementing Unity Analytics and stores these data internally in an aggregated and anonymous form. These data are capable of describing the total amount of playtime taking place in each product that implements Unity Analytics for each day of the year within each of the 250 territories worldwide. The raw data, consisting of day-by-day playtime logs, contained the following variables (data features): (1) a unique but anonymized identifier for a user, (2) an identifier for the country in which a user’s play took place, and (3) the total duration of playtime on that day for that user. These data were aggregated by us at a country level; in essence, for every day from January 1, 2020, to December 5, 2021, we summed the total duration of play for every player within Unity’s data grouped within each individual country. These dates were selected for analysis as January 1, 2020, is the first date on which any containment or closure policies were recorded for any country within the OxCGRT database, and this research was conducted soon after December 5, 2021, so this was the most recent date for which Unity Technologies had data stored in their warehouses at the time of analysis.

Data describing the containment and closure policies used by governments worldwide were taken from the publicly available data sets provided by the OxCGRT [5]. The OxCGRT provides daily estimates of containment and closure policies for 185 individual countries during each of the dates spanning January 1, 2020, onward; as our playtime data covered January 1, 2020, to December 5, 2021, we used the same range of dates in the OxCGRT data for this study. The OxCGRT tracks the enforcement level and geographic scope of 8 separate containment and closure policies in each of the countries in its database via the use of an ordinal scale. These individual policies are as follows:

1. School closures: the degree to which policy requires schools and universities to be closed within a territory. This policy category ranges from 0 (no measures in place) to 3 (requires closing all levels of schools). Intermediate enforcement levels are 1 (recommendation that schools close or schools are open but with significant alterations) and 2 (some subset of schools is required to be closed).
2. Workplace closures: the degree to which policy requires workplaces to be closed within a territory. This policy category ranges from 0 (no measures in place) to 3 (workplace closure or work from home is required for all-but-essential workplaces). Intermediate enforcement levels are 1 (workplace closure is recommended or all businesses are open with significant alterations) and 2 (workplaces are required to be closed for some sectors or categories of workers).
3. Cancellation of public events: the degree to which policy requires public events to be cancelled. This policy category ranges from 0 (no measures in place) to 2 (requires cancelling public events). An intermediate enforcement level is recorded as 1 (recommends cancelling public events).
4. Restrictions on gatherings: the degree to which policy requires gatherings with specific numbers of people to be limited in some way. This policy category ranges from 0 (no measures in place) to 4 (restrictions on gatherings of ≤10 people). Intermediate enforcement levels are as follows: 1 (restrictions on gatherings of >1000 people), 2 (restrictions on gatherings of 101-1000 people), and 3 (restrictions on gatherings of 11-100 people).
5. Closure of public transport: the degree to which public transport closures are required or recommended by policy. This policy category ranges from 0 (no measures in place) to 2 (requires the closure of public transport or prohibits the use of public transport for most citizens). Intermediate level (1) denotes a situation in which the government recommends the closure of public transport or significantly reduces the volume or routes or means of public transport that are available.
6. Stay-at-home requirements: the degree to which policy requires individuals to be confined to their homes or “shelter in place” requirements are in force. This policy category ranges from 0 (no measures in place) to 3 (a situation in which individuals are required not to leave the house with minimal exceptions). Intermediate enforcement categories are as follows: 1 (recommendations not to leave the house) and 2 (a requirement not to leave the house with exceptions for exercise, grocery shopping, and “essential” activities).
7. Restrictions on internal movement: the degree to which policy restricts movement between cities or regions within a territory. This policy category ranges from 0 (no measures in place) to 2 (some form of restriction on internal movements in place). An intermediate category of 1 represents a situation in which a recommendation not to travel between regions or cities is made.
8. International travel controls: the degree to which policy restricts movement from or to outside a territory. This policy category ranges from 0 (no restrictions) to 4 (ban on travel to or from all regions or total border closure). The intermediate enforcement categories consist of 1 (screening arrivals), 2 (quarantining arrivals from some or all regions), and 3 (banning arrivals from some or all regions).

Raw data from the OxCGRT database comprised daily estimates of the enforcement level of the aforementioned containment and closure policies for 185 individual countries during each of the dates spanning January 1, 2020, to December 5, 2021. In addition, each enforcement level for each containment or closure policy was annotated with a flag describing its geographical scope: whether a policy was applied generally within a territory or whether it was enforced at a targeted level. To make as few assumptions as possible regarding the differences between these enforcement levels, in all our analyses, each policy was treated as a nominal variable with 2*n* – 1 levels, where *n* is the number of enforcement categories within that variable. For example, *cancellation of public events* was treated as a nominal variable with 5 levels (1: no restrictions; 2: recommendation to cancel public events, targeted at the regional level; 3: general recommendation to cancel public events within a country; 4: requirement to cancel public events, targeted at the regional level; and 5: general requirement to cancel public events within a country). As another example, in Japan, on August 5, 2021, some regions of the country recommended cancelling public events (level 2 of the aforementioned nominal variable); on August 6, 2021, such recommendations became nationwide (level 3 of the aforementioned nominal variable).

The data used in this study were obtained from the following territories: Afghanistan, Albania, Algeria, Andorra, Angola, Argentina, Aruba, Australia, Austria, Azerbaijan, Bahamas, Bahrain, Bangladesh, Barbados, Belarus, Belgium, Belize, Benin, Bermuda, Bhutan, Bolivia, Bosnia and Herzegovina, Botswana, Brazil, Brunei, Bulgaria, Burkina Faso, Burundi, Cambodia, Cameroon, Canada, Cape Verde, Central African Republic, Chad, Chile, China, Colombia, Comoros, Congo, Costa Rica, Côte d'Ivoire, Croatia, Cyprus, Czech Republic, Democratic Republic of Congo, Denmark, Djibouti, Dominica, Dominican Republic, Ecuador, Egypt, El Salvador, Eritrea, Estonia, Eswatini, Ethiopia, Faeroe Islands, Fiji, Finland, France, Gabon, Gambia, Georgia, Germany, Ghana, Greece, Greenland, Guam, Guatemala, Guinea, Guyana, Haiti, Honduras, Hong Kong, Hungary, Iceland, India, Indonesia, Iran, Iraq, Ireland, Israel, Italy, Jamaica, Japan, Jordan, Kazakhstan, Kenya, Kiribati, Kosovo, Kuwait, Kyrgyz Republic, Laos, Latvia, Lebanon, Lesotho, Liberia, Libya, Liechtenstein, Lithuania, Luxembourg, Macao, Madagascar, Malawi, Malaysia, Mali, Malta, Mauritania, Mauritius, Mexico, Moldova, Monaco, Mongolia, Morocco, Mozambique, Myanmar, Nepal, Netherlands, New Zealand, Nicaragua, Niger, Nigeria, Norway, Oman, Pakistan, Palestine, Panama, Papua New Guinea, Paraguay, Peru, Philippines, Poland, Portugal, Puerto Rico, Qatar, Romania, Russia, Rwanda, San Marino, Saudi Arabia, Senegal, Serbia, Seychelles, Sierra Leone, Singapore, Slovak Republic, Slovenia, Solomon Islands, Somalia, South Africa, South Korea, South Sudan, Spain, Sri Lanka, Sudan, Suriname, Sweden, Switzerland, Syria, Taiwan, Tajikistan, Tanzania, Thailand, Timor-Leste, Togo, Tonga, Trinidad and Tobago, Tunisia, Turkey, Turkmenistan, Uganda, Ukraine, United Arab Emirates, United Kingdom, United States, United States Virgin Islands, Uruguay, Uzbekistan, Vanuatu, Venezuela, Vietnam, Yemen, Zambia, and Zimbabwe.

### Data Limitations

The work presented in this paper relies on the analysis of data from video games created with or using components of the Unity game engine, where Unity Analytics is enabled. Although there is no obvious reason why games made using the Unity engine would not be representative, there is no direct way to verify such an assumption. In addition, the user accounts in the data under analysis are specific to individual products made using Unity Analytics; for example, if one human being were to play 2 different games, they would be represented in our data as 2 separate user accounts. Thus, our data are unable to model phenomena in which individuals cycle between multiple separate games.

### Data Transformation

The countries in our data set varied widely in terms of playtime. China, for example, had a total of 36,538,076,867 hours of playtime during the period in question, whereas Yemen had >421 times less (86,703,378 hours in total). Therefore, to measure the relative impact of containment and closure policies within a country, daily averages of playtime were scaled within each country’s data such that each country’s overall playtime per day had an SD of 1 (and a mean of 0), and random intercepts dropped from each model as within-group means were centered at 0. This is in line with recommendations for interpreting within-group variation in multilevel modeling [43]. No other transformations were applied to our data.

### Modeling Approach

To investigate the impact of specific policy decisions on playtime, we fitted a series of 3 separate multilevel models to data on playtime from the 184 countries in our data set. Our first model estimated the ability of policy decisions to explain the variance in playtime per user (ie, how many hours individual gamers tend to play a game each day). Our second and third models expanded on this relationship by estimating the ability of policy decisions to explain the variance in the number of daily unique users within a country and the total amount of playtime within that country. To render the analyses tractable, the data were first aggregated at weekly levels before analysis.

Apart from their outcome variables (playtime per user, number of users, and total playtime), our models were otherwise identical—each contained fixed effects that allowed for modeling of the impact of the differing enforcement levels and geographical scopes of the 8 containment and closure policy categories within the OxCGRT database [5]. The policy categories were school closures, workplace closures, the cancellation of public events, restrictions on gatherings, closure of public transport, stay-at-home requirements, restrictions on internal movement, and international travel controls. These analyses accounted for both the geographical scope of a policy (targeted to a specific area or generally enforced within a territory) and the enforcement severity of that policy (on an ordinal scale ranging from 1 to 4).

**Figure 1:**
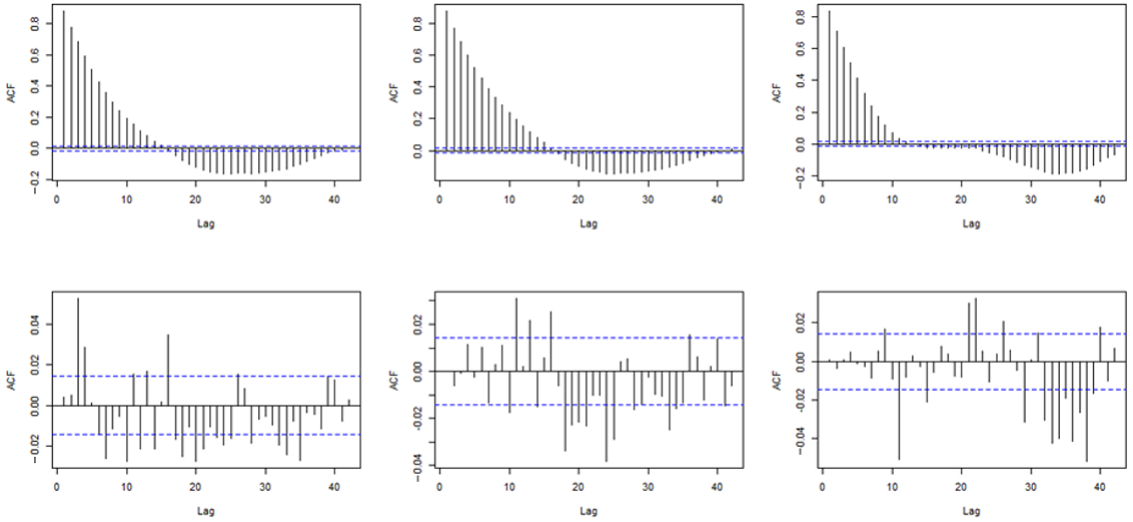
Autocorrelation function (ACF) plots. The top row represents the ACF of residuals in uncorrected models (left to right: total playtime, playtime per user, and number of users). The bottom row represents the ACF of corrected models (left to right: total playtime, playtime per user, and number of users). The dotted lines represent significance bounds.

To account for subnational linear temporal trends in playtime, we also included random intercepts and random country × week slopes within our models. To account for temporal dependency in model residuals, we corrected for any autoregressive–moving-average (ARMA) process that may appear within these residuals [44,45]. An uncorrected multilevel model was initially produced for each of our outcome variables. The order of any (p,q) ARMA structure within the residuals of these 3 separate models was algorithmically estimated using the Hyndman-Khandakar procedure [46]. This suggested that an ARMA(1) process would be able to account for autocorrelation in the residuals of the model that dealt with total playtime, an ARMA(2,3) process would be able to account for autocorrelation in the residuals of the model that dealt with the daily number of users, and an ARMA(1,5) process would be able to account for autocorrelation in the residuals of the playtime per user model. The models were then rebuilt with ARMA errors of the orders described previously, resulting in substantially reduced autocorrelation in error terms (Figure 1).

**Figure 2:**
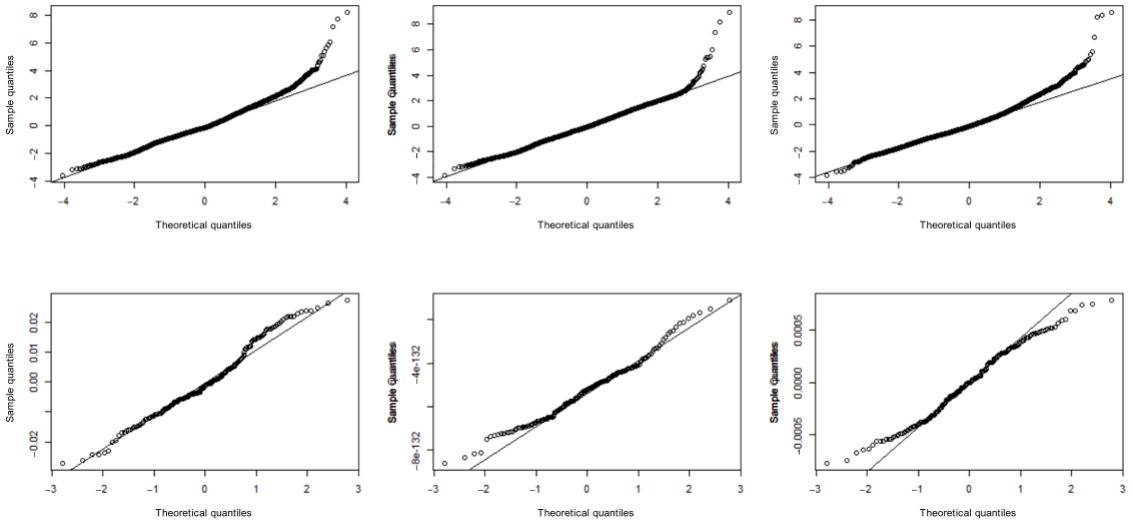
Q-Q plots of model residuals. The top row represents overall residuals (from left to right: total playtime, number of users, and playtime per user). The bottom row represents the residuals associated with the random slopes.

Multicollinearity within all models was assessed via inspection of variance inflation factor statistics. No variance inflation factor was >5, suggesting an absence of evidence for significant multicollinearity issues within each model. Random slopes in all models appeared approximately normally distributed; a *Q*-*Q* plot suggested that the residuals associated with each overall model were also approximately normal, albeit with a slightly heavy upper tail (Figure 2).

## Results

### Overview

**Figure 3:**
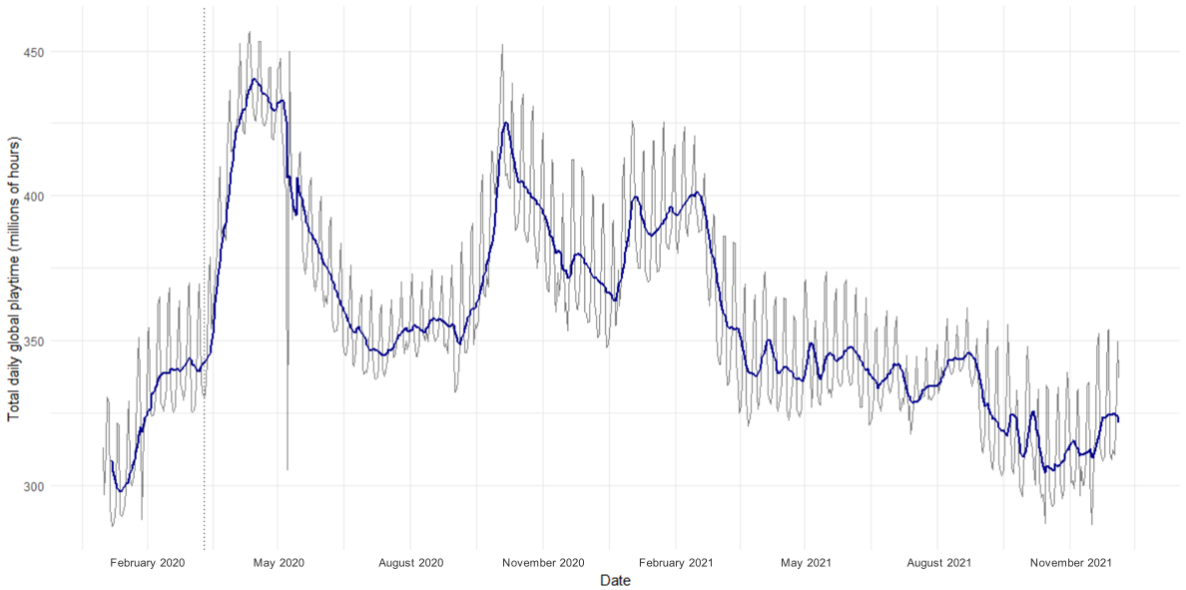
Total global daily playtime from January 1, 2020, to December 5, 2021. The solid blue line represents a weekly simple moving average. The dotted line represents the World Health Organization declaration of the pandemic on March 11, 2020.

**Figure 4:**
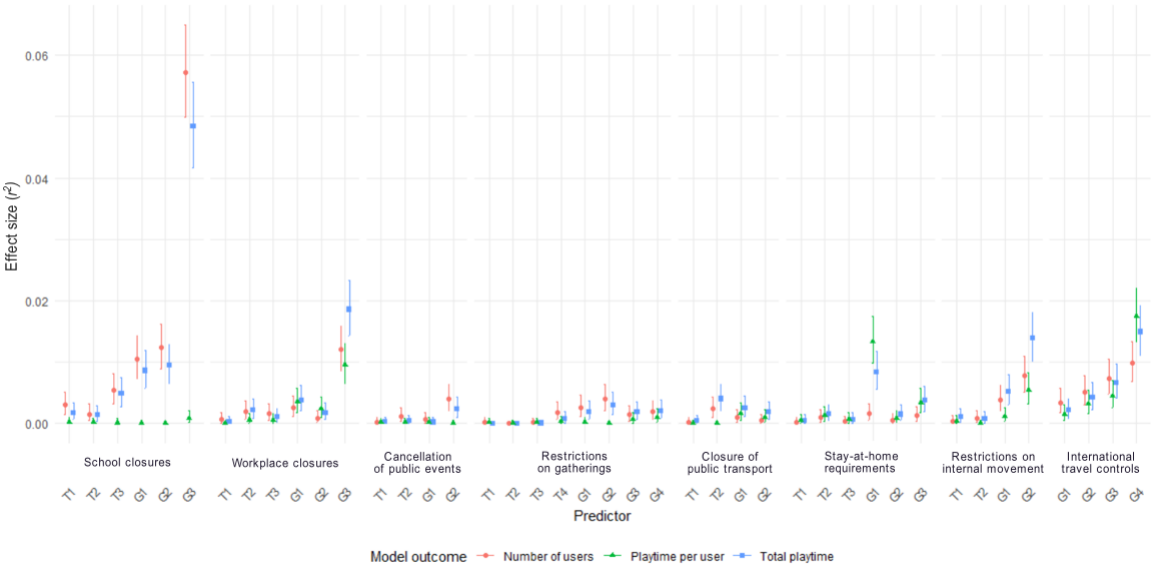
All associations between containment and closure policies and outcomes for the 3 models. The error bars represent the 95% CIs of the effect size associated with each policy decision. The letters G and T in a policy’s annotation refer to whether that policy’s geographical scope was general or targeted. The numbers (1-4) represent the enforcement level of that policy (see the Methods section for more details).

Overall, a sum total of 251.88 billion hours of playtime were recorded during the period under analysis (January 1, 2020, to December 5, 2021), equating to an average global daily playtime of 357.28 million hours. Global playtime varied significantly during the time under analysis (Figure 3). Figure 4 summarizes the outcomes of multilevel modeling, which are described in the following sections.

### Multilevel Modeling

The entirety of each of our 3 multilevel models, including the estimated relationship between each enforcement level and the geographical scope of each policy decision, is reported in Tables 1-3.

**Table 1:** Daily playtime per user—the impact of each enforcement level and geographical scope of the 8 containment and closure policies. Unstandardized coefficients are given, along with t statistics and an effect size estimate. Transformation before analysis means that each β coefficient equates to the increase or decrease in daily playtime per user associated with a specific enforcement level of a specific policy within an average country, measured in SDs.

Policy, scope, and level				β (95% CI)		*t* test (*df*)		Effect size (95% CI)
**School closures**								
	**Targeted**							
		1	−.061 (−.201 to .079)		−0.857		<0.001 (<0.001 to <0.001)	
		2	.025 (−.051 to .100)		0.639		<0.001 (<0.001 to <0.001)	
		3	−.002 (−.076 to .072)		−0.054		<0.001 (<0.001 to <0.001)	
	**General**							
		1	.018 (−.038 to .074)		0.618		<0.001 (<0.001 to <0.001)	
		2	.010 (−.051 to .071)		0.329		<0.001 (<0.001 to <0.001)	
		3	.051 (−.008 to .109)		1.705		<0.001 (<0.001 to 0.002)	
**Workplace closures**								
	**Targeted**							
		1	.003 (−.099 to .106)		0.064		<0.001 (<0.001 to <0.001)	
		2	.051 (−.022 to .125)		1.366		<0.001 (<0.001 to 0.002)	
		3	.137 (.053 to .221)		3.205^a^		0.002 (<0.001 to 0.004)	
	**General**							
		1	.040 (−.018 to .099)		1.343		<0.001 (<0.001 to 0.002)	
		2	.099 (.040 to .157)		3.295^b^		0.003 (0.002 to 0.006)	
		3	.244 (.169 to .319)		6.396^b^		0.010 (0.007 to 0.013)	
**Cancellation of public events**								
	**Targeted**							
		1	−.042 (−.154 to .070)		−0.733		<0.001 (<0.001 to <0.001)	
		2	−.038 (−.116 to .040)		−0.948		<0.001 (<0.001 to <0.001)	
	**General**							
		1	−.023 (−.085 to .039)		−0.725		<0.001 (<0.001 to <0.001)	
		2	−.010 (−.073 to .053)		−0.3		<0.001 (<0.001 to <0.001)	
**Restrictions on gatherings**								
	**Targeted**							
		1	−.146 (−.427 to .134)		−1.023		<0.001 (<0.001 to <0.001)	
		2	−.066 (−.201 to .069)		−0.958		<0.001 (<0.001 to <0.001)	
		3	.041 (−.049 to .130)		0.891		<0.001 (<0.001 to <0.001)	
		4	.062 (−.022 to .146)		1.444		<0.001 (<0.001 to 0.002)	
	**General**							
		1	−.011 (−.110 to .088)		−0.214		<0.001 (<0.001 to <0.001)	
		2	−.036 (−.108 to .036)		−0.97		<0.001 (<0.001 to 0.001)	
		3	−.004 (−.069 to .060)		−0.133		<0.001 (<0.001 to <0.001)	
		4	.062 (−.006 to .130)		1.775		0.001 (<0.001 to 0.002)	
**Closure of public transport**								
	**Targeted**							
		1	−.010 (−.090 to .070)		−0.236		<0.001 (<0.001 to <0.001)	
		2	.091 (.016 to .165)		2.391^c^		0.002 (<0.001 to 0.003)	
	**General**							
		1	.002 (−.042 to .045)		0.074		<0.001 (<0.001 to <0.001)	
		2	.065 (−.003 to .133)		1.876		<0.001 (<0.001 to 0.002)	
**Stay-at-home requirements**								
	**Targeted**							
		1	.060 (−.026 to .147)		1.361		<0.001 (<0.001 to 0.001)	
		2	.046 (−.014 to .106)		1.491		<0.001 (<0.001 to 0.002)	
		3	.095 (−.006 to .195)		1.847		<0.001 (<0.001 to 0.002)	
	**General**							
		1	.047 (<−.001 to .095)		1.93		0.001 (<0.001 to 0.003)	
		2	.169 (.115 to .223)		6.103^b^		0.013 (0.010 to 0.017)	
		3	.254 (.137 to .372)		4.231^b^		0.003 (0.002 to 0.006)	
**Restrictions on internal movement**								
	**Targeted**							
		1	.047 (−.035 to .130)		1.126		<0.001 (<0.001 to 0.001)	
		2	.053 (.002 to .105)		2.022^c^		0.001 (<0.001 to 0.003)	
	**General**							
		1	.006 (−.043 to .054)		0.241		<0.001 (<0.001 to <0.001)	
		2	.117 (.065 to .169)		4.382^b^		0.005 (0.003 to 0.008)	
**International travel controls**								
	**General**							
		1	.084 (.012 to .157)		2.280^c^		0.002 (<0.001 to 0.003)	
		2	.122 (.049 to .196)		3.266^a^		0.003 (0.002 to 0.005)	
		3	.141 (.068 to .214)		3.795^b^		0.005 (0.003 to 0.007)	
		4	.283 (.204 to .362)		7.045^b^		0.017 (0.013 to 0.022)	
Week (scaled 0-1)				.085 (−.130 to −.040)		−3.709^b^		0.022 (0.018 to 0.027)

^a^*P*<.01.

^b^*P*<.001.

^c^*P*<.05.

**Table 2:** Daily number of users—impact of each enforcement level and geographical scope of the 8 containment and closure policies. Unstandardized coefficients are given, along with t statistics and an effect size estimate. Transformation before analysis means that each β coefficient equates to the increase or decrease in daily number of users associated with a specific enforcement level of a specific policy within an average country, measured in SDs.

Policy, scope, and level				β (95% CI)		*t* test (*df*)		Effect size (95% CI)
**School closures**								
	**Targeted**							
		1	.190 (.081 to .299)		3.416^a^		0.003 (0.001 to 0.005)	
		2	.132 (.073 to .192)		4.381^a^		0.005 (0.003 to 0.008)	
		3	.210 (.153 to .267)		7.187^a^		0.012 (0.009 to 0.016)	
	**General**							
		1	.046 (.002 to .090)		2.027^b^		0.002 (<0.001 to 0.003)	
		2	.142 (.095 to .190)		5.831^a^		0.011 (0.007 to 0.014)	
		3	.343 (.297 to .388)		14.760^a^		0.057 (0.050 to 0.065)	
**Workplace closures**								
	**Targeted**							
		1	.073 (−.006 to .152)		1.802		<0.001 (<0.001 to 0.002)	
		2	.071 (.014 to .129)		2.420^b^		0.002 (<0.001 to 0.003)	
		3	.063 (−.002 to .128)		1.888		<0.001 (<0.001 to 0.002)	
	**General**							
		1	.057 (.011 to .103)		2.416^b^		0.002 (<0.001 to 0.004)	
		2	.066 (.020 to .112)		2.794^c^		0.003 (0.001 to 0.004)	
		3	.214 (.156 to .273)		7.222^a^		0.012 (0.009 to 0.016)	
**Cancellation of public events**								
	**Targeted**							
		1	−.044 (−.132 to .043)		−1.001		<0.001 (<0.001 to <0.001)	
		2	.052 (−.009 to .113)		1.664		<0.001 (<0.001 to 0.002)	
	**General**							
		1	.050 (.001 to .099)		2.013^b^		0.001 (<0.001 to 0.003)	
		2	.100 (.051 to .149)		4.001^a^		0.004 (0.002 to 0.006)	
**Restrictions on gatherings**								
	**Targeted**							
		1	.128 (−.088 to .345)		1.161		<0.001 (<0.001 to <0.001)	
		2	.044 (−.061 to .148)		0.819		<0.001 (<0.001 to <0.001)	
		3	.116 (.046 to .186)		3.263^c^		0.003 (0.001 to 0.005)	
		4	.074 (.008 to .139)		2.201^b^		0.001 (<0.001 to 0.003)	
	**General**							
		1	.003 (−.074 to .080)		0.08		<0.001 (<0.001 to <0.001)	
		2	.069 (.013 to .126)		2.395^b^		0.002 (<0.001 to 0.004)	
		3	.090 (.040 to .141)		3.495^a^		0.004 (0.002 to 0.006)	
		4	.066 (.012 to .119)		2.401^b^		0.002 (<0.001 to 0.004)	
**Closure of public transport**								
	**Targeted**							
		1	.026 (−.038 to .089)		0.799		<0.001 (<0.001 to 0.001)	
		2	.053 (−.006 to .111)		1.774		<0.001 (<0.001 to 0.002)	
	**General**							
		1	.043 (.009 to .078)		2.448^b^		0.002 (0.001 to 0.004)	
		2	.036 (−.017 to .089)		1.346		<0.001 (<0.001 to 0.001)	
**Stay-at-home requirements**								
	**Targeted**							
		1	.031 (−.037 to .098)		0.886		<0.001 (<0.001 to <0.001)	
		2	.024 (−.024 to .071)		0.987		<0.001 (<0.001 to 0.001)	
		3	.060 (−.019 to .138)		1.496		<0.001 (<0.001 to 0.002)	
	**General**							
		1	.032 (−.006 to .069)		1.666		<0.001 (<0.001 to 0.002)	
		2	.045 (.002 to .088)		2.056^b^		0.002 (<0.001 to 0.003)	
		3	.119 (.029 to .210)		2.575^b^		0.001 (<0.001 to 0.003)	
**Restrictions on internal movement**								
	**Targeted**							
		1	.038 (−.027 to .103)		1.156		<0.001 (<0.001 to 0.001)	
		2	.075 (.035 to .115)		3.639^a^		0.004 (0.002 to 0.006)	
	**General**							
		1	.034 (−.004 to .072)		1.747		<0.001 (<0.001 to 0.002)	
		2	.109 (.068 to .150)		5.218^a^		0.008 (0.005 to 0.011)	
**International travel controls**								
	**General**							
		1	.099 (.042 to .156)		3.418^a^		0.003 (0.002 to 0.006)	
		2	.122 (.064 to .180)		4.140^a^		0.005 (0.003 to 0.008)	
		3	.141 (.084 to .199)		4.819^a^		0.007 (0.005 to 0.011)	
		4	.166 (.103 to .228)		5.210^a^		0.010 (0.007 to 0.013)	
Week (scaled 0-1)				.314 (.265 to .362)		12.738^a^		0.340 (0.328 to 0.352)

^a^*P*<.001.

^b^*P*<.05.

^c^*P*<.01.

**Table 3:** Total playtime—impact of each enforcement level and geographical scope of the 8 containment and closure policies. Unstandardized coefficients are given, along with t statistics and an effect size estimate. Transformation before analysis means that each β coefficient equates to increase or decrease in total playtime associated with a specific enforcement level of a specific policy within an average country, measured in SDs.

Policy, scope, and level				β (95% CI)		*t* test (*df*)		Effect size (95% CI)
**School closures**								
	**Targeted**							
		1	.141 (.033 to .248)		2.569^a^		0.002 (<0.001 to 0.003)	
		2	.124 (.065 to .182)		4.152^b^		0.005 (0.003 to 0.007)	
		3	.181 (.124 to .237)		6.263^b^		0.009 (0.006 to 0.013)	
	**General**							
		1	.043 (<−.001 to .087)		1.934		0.001 (<0.001 to 0.003)	
		2	.126 (.079 to .174)		5.241^b^		0.009 (0.006 to 0.012)	
		3	.309 (.264 to .354)		13.494^b^		0.048 (0.042 to 0.056)	
**Workplace closures**								
	**Targeted**							
		1	.046 (−.032 to .125)		1.152		<0.001 (<0.001 to 0.001)	
		2	.056 (<−.001 to .113)		1.943		0.001 (<0.001 to 0.002)	
		3	.091 (.026 to .155)		2.766^c^		0.002 (<0.001 to 0.003)	
	**General**							
		1	.060 (.014 to .105)		2.574^a^		0.002 (<0.001 to 0.004)	
		2	.080 (.034 to .125)		3.436^b^		0.004 (0.002 to 0.006)	
		3	.263 (.206 to .321)		8.978^b^		0.019 (0.014 to 0.023)	
**Cancellation of public events**								
	**Targeted**							
		1	−.044 (−.130 to .042)		−0.993		<0.001 (<0.001 to <0.001)	
		2	.027 (−.033 to .087)		0.886		<0.001 (<0.001 to <0.001)	
	**General**							
		1	.029 (−.019 to .077)		1.195		<0.001 (<0.001 to 0.001)	
		2	.076 (.028 to .125)		3.080^c^		0.002 (0.001 to 0.004)	
**Restrictions on gatherings**								
	**Targeted**							
		1	.024 (−.190 to .239)		0.221		<0.001 (<0.001 to <0.001)	
		2	.026 (−.077 to .128)		0.488		<0.001 (<0.001 to <0.001)	
		3	.097 (.028 to .166)		2.760^c^		0.002 (<0.001 to 0.004)	
		4	.083 (.019 to .148)		2.526^a^		0.002 (<0.001 to 0.004)	
	**General**							
		1	−.002 (−.078 to .074)		−0.055		<0.001 (<0.001 to <0.001)	
		2	.043 (−.013 to .099)		1.504		<0.001 (<0.001 to 0.002)	
		3	.076 (.026 to .126)		2.983^c^		0.003 (0.001 to 0.005)	
		4	.068 (.015 to .121)		2.504^a^		0.002 (<0.001 to 0.004)	
**Closure of public transport**								
	**Targeted**							
		1	.033 (−.029 to .096)		1.042		<0.001 (<0.001 to 0.001)	
		2	.086 (.029 to .144)		2.931^c^		0.003 (0.001 to 0.004)	
	**General**							
		1	.055 (.021 to .089)		3.152^c^		0.004 (0.002 to 0.006)	
		2	.070 (.017 to .122)		2.610^c^		0.002 (<0.001 to 0.004)	
**Stay-at-home requirements**								
	**Targeted**							
		1	.045 (−.022 to .112)		1.321		<0.001 (<0.001 to 0.001)	
		2	.035 (−.012 to .081)		1.447		<0.001 (<0.001 to 0.002)	
		3	.094 (.017 to .172)		2.392^a^		0.001 (<0.001 to 0.003)	
	**General**							
		1	.039 (.002 to .076)		2.077^a^		0.001 (<0.001 to 0.003)	
		2	.103 (.061 to .145)		4.766^b^		0.008 (0.006 to 0.012)	
		3	.206 (.116 to .295)		4.484^b^		0.004 (0.002 to 0.006)	
**Restrictions on internal movement**								
	**Targeted**							
		1	.062 (−.002 to .126)		1.898		0.001 (<0.001 to 0.002)	
		2	.086 (.046 to .126)		4.234^b^		0.005 (0.003 to 0.008)	
	**General**							
		1	.031 (−.006 to .069)		1.634		<0.001 (<0.001 to 0.002)	
		2	.144 (.103 to .184)		6.972^b^		0.014 (0.010 to 0.018)	
**International travel controls**								
	**General**							
		1	.078 (.022 to .134)		2.711^c^		0.002 (<0.001 to 0.004)	
		2	.108 (.051 to .165)		3.718^b^		0.004 (0.002 to 0.007)	
		3	.132 (.075 to .189)		4.552^b^		0.007 (0.004 to 0.010)	
		4	.201 (.140 to .263)		6.418^b^		0.015 (0.011 to 0.019)	
Week (scaled 0-1)				.269 (.221 to .317)		10.983^b^		0.281 (0.269 to 0.293)

^a^*P*<.05.

^b^*P*<.001.

^c^*P*<.01.

First, we investigated the ability of containment and closure policies to explain variations in playtime per user. This analysis was of particular importance in the context of the literature, which frequently deals with the idea that lockdown policies may lead to individual gamers tending to spend substantially longer in-game. Our overall model was able to explain 24.9% (*r*^2^: 95% CI 0.239-0.263) of the variance in playtime per user. Crucially, no policy decision was able to explain variance in excess of an *r*^2^ of 0.04 in mean duration of play per user; widely used guidelines for practically meaningful effect sizes in media effects and behavioral science research place the smallest effect size of importance at *r*^2^≥0.04 [47]. Only 2 policy decisions were able to explain >1% of variance in playtime per user. These were the general requirement that a country’s borders close (*r*^2^=0.017, 95% CI 0.013-0.022) and the general requirement that people not leave their houses except for groceries, exercise, and essential activities (*r*^2^=0.013, 95% CI 0.010-0.013). Notedly, no degree of school closure was able to explain a practically meaningful amount of variation in the average amount of playtime per user (*r*^2^ values for each of these variables were measured as <0.001). The full model is presented in Table 1.

We then analyzed the total amount of playtime within a territory. Fixed effects were able to explain 61.3% (*r*^2^: 95% CI 0.604-0.621) of the variance in total playtime within the territories under analysis. The only policy decision able to explain variance in playtime of plausibly meaningful magnitude using this guideline was the general requirement that all schools within a territory close. This policy decision was associated with significantly higher amounts of playtime (*r*^2^=0.048, 95% CI 0.042-0.056; *P*<.001). The full model is presented in Table 2.

Finally, we attempted to understand the roots of this change in playtime by investigating whether containment and closure policies were associated with increased numbers of unique game users. A multilevel model again was able to explain most of the variance in daily numbers of users within the territories under analysis (*r*^2^=0.628, 95% CI 0.620-0.636). However, again, the only policy decision able to explain a variance of *r*^2^≥0.04 was the general requirement that all schools within a territory close, which led to significantly higher numbers of video game users (*r*^2^=0.057, 95% CI 0.050-0.065; *P*<.001). Apart from this, only 3 other policy decisions were able to explain >1% of variance in the number of daily unique users playing games. Notably, all these policy decisions were related to the closure of schools or workplaces. They comprised the requirement that a targeted subset of schools within a territory close (*r*^2^=0.012, 95% CI 0.009-0.016), the general requirement that all nonessential workplaces within a territory close (*r*^2^=0.012, 95% CI 0.009-0.016; *P*<.001), and the recommendation (rather than requirement) that all schools within a territory close (*r*^2^=0.011, 95% CI 0.007-0.014). The full model is presented in Table 3.

The impacts of all the aforementioned policies were positive—they were associated with increases in the outcome measure of each individual model, and no policies were associated with statistically significant decreases in any outcome. Random slopes had very small SDs (<0.001), indicating minimal differences in temporal trends in gaming when other (fixed) factors were accounted for.

## Discussion

### Principal Findings

Substantial attention has been paid to the idea that lockdown policies may have created socially meaningful changes in gaming. Theory in this domain ranges from speculation that gamers may play more during the enforcement of social restrictions to fulfill their relatedness needs to concerns that the closure of schools may lead to heavy and excessive gaming among young people [17,29,34,35].

However, our findings do not support either of these theoretical positions. All levels of the 8 containment and closure policies measured in this study had little impact on the average duration of playtime per game per gamer. Notably, restrictions on social gatherings failed to explain even a small amount of variance (*r*^2^<0.01 in all cases) in either the number of players or the amount of time that gamers spent in-game. This contrasts with speculation that increases in gaming during the pandemic may have been driven primarily by a need to socialize.

Indeed, by far the largest association between policy and gaming observed in this study involved the complete closure of schools within a territory. This was associated with an increase in the number of individuals playing games and an increase in total playtime within a territory (Tables 2 and 3). However, crucially, school closures were not related to increases in the average amount of time that each observed user tended to spend in-game (Table 1). Thus, our work fails to substantiate concerns that lockdowns caused children and adolescents to play games more heavily while simultaneously aligning with industry reports that playtime increased during the pandemic. Indeed, all geographical scopes and enforcement levels of school closures were unable to explain even 0.01% of the variance in duration of play per user. This observation—that global playtime may have increased during lockdowns whereas *play per player* did not—provides an important novel addition to the debate over the impact of the pandemic on gaming.

Indeed, it is interesting to note that these results contrast sharply with the results of several self-report studies within the field; previous studies investigating the impact of lockdowns on gaming have found that young people report playing games more heavily during lockdowns, which would stand in stark contrast to our results [17,29]. However, it is important to note the differing perspective that our telemetry data afford in this case—those studies were forced to rely on single-country convenience samples and self-report of recollected playtime; the discrepancy between the results reported in this paper and prior work may be due to our use of global behavioral data. Another explanation is that individuals who played for longer tended to play outside Unity Analytics–enabled mobile and desktop games. This play may have happened, for example, exclusively in console games, which were not tracked in this study.

Thus, a primary contribution of this study is negative in nature. In contrast to arguments that lockdowns may commonly lead to important upticks in excessive play, only 2 policy decisions were able to explain even small amounts of variance in daily playtime per user. The largest of these was associated with the complete closure of a country’s borders—when countries completely closed their borders, individuals tended to play for longer periods. This association has not been speculated about in the academic literature. In addition to international travel controls, it is important to note that some implementations of workplace closures, stay-at-home requirements, and restrictions on internal movement were also associated with modest increases in playtime per user. What might have underpinned each of these changes? A plausible explanation for this is the use of games as a substitution activity taking the place of, for example, a foreign vacation when that vacation becomes inaccessible. One could also imagine stay-at-home requirements, the sudden implementation of widespread working from home, and strict restrictions on travel within a country bearing similar effects. However, it is important to note that, in each of these cases, the effects are small in magnitude and may not bear any practical significance; the only observed effects that exceeded common cutoffs (*r*^2^≥0.04) in the social science literature for statistically important effects were those associated with the complete closure of schools within a territory [47]. What might have led to these increases? As hinted at throughout the Introduction section, there is a host of social factors that may explain these associations. For example, a lack of oversight associated with being physically present in schools may have afforded young people additional opportunities to take part in their hobbies, parents may have made use of games as a “digital babysitter” to cope with the pressures associated with having children at home, or young people may simply have used video games as a form of stress relief to deal with the turbulent times associated with their schools being closed down. Unpicking exactly why the associations observed in this study actually occurred should be a target for significant future work.

Finally, it is important to consider that, although the mean duration of play per user may be unaffected by lockdown policies, other socially important changes in the distribution of play may nonetheless be associated with these decisions. For example, it is plausible that school closures led to the influx of a large group of casual players with low daily playtime at the same time as a small group of gamers began to engage very heavily in-game and developed a very high daily average playtime. This kind of distributional shift is beyond the scope of this work but should be an important focus for future research.

### Limitations

A limitation of the approach taken in this study is its generalized nature. In this study, we sought to examine whether the implementation of various containment and closure policies was associated *in general* with changes in 3 crucial playtime-related variables. However, by doing so, we naturally overlooked the potential for local effects. It may be the case that containment and closure policies *within specific countries or regions* were associated with reductions in playtime variables even if overall there was no global effect. Although misaligned with the core research questions that we were investigating (which were about the effects of policies in general), these are nonetheless interesting from both a theoretical and a public health perspective. If specific policies afford differential impacts in different parts of the world, the evidence base must incorporate this knowledge, for example, for maximal pandemic preparedness. However, it is important to point out that this represents the first time that behavioral data have been used to model the impact of different policies on gaming during the COVID-19 pandemic. Thus, it is reasonable to suggest that not all possible analyses will have been undertaken in this study—some, such as those regarding the issues outlined in this paragraph, must remain priorities for future research.

Thus, this study’s use of behavioral data affords unique insights into how gaming changed during the COVID-19 pandemic. However, it is important to contextualize these findings within several key limitations regarding the data source used in this study.

To begin with, this study exclusively used data from mobile and desktop games and did not contain any data from the console market. When one considers potential differences in interaction styles between these domains, it becomes plausible that an effect of lockdowns may exist in the console domain but not in the desktop or mobile markets. For example, one can imagine that increases in very heavy play may occur primarily in console games, where individuals primarily interact using ergonomically designed gamepads, and not in desktop or mobile games, where this is not the case. However, such differences may be overstated. Research, for example, has found little difference in player reports of comfort between keyboard-based setups and gamepads [48]. It is unclear from the current evidence base whether excessive play primarily takes place on desktop, console, or any other device.

Furthermore, as noted elsewhere, console gaming may not occupy a large market share of playtime when it comes to specific demographics such as young people [29]. Second, the individual identifiers used in this study operated on an account level rather than a platform level. Therefore, they could not identify situations in which one individual migrated between games. Thus, changes in the total number of users cannot truly differentiate between a scenario in which many new gamers entered the market and one in which extant users began switching rapidly between multiple different games. It is our opinion that the former explanation is more plausible—we have no previous theory that informs us of why schools locking down would cause gamers to start rapidly switching between games, but we do have a previous theory that informs us of why schools locking down would lead to more individuals engaging in gaming. However, this limitation must be the target of significant future research.

Finally, the data used in this study were drawn from one stakeholder, Unity Technologies. Unity’s analytical solutions underpin a large proportion of the gaming market. They have afforded us access to an unprecedentedly large sample of player behavior—>250 billion hours of playtime. However, they do not allow us to measure playtime in *every single game in existence*. Thus, it is possible that some effect may exist in a specific product or set of products for which we did not have access to data—*Fortnite*, for example, was not developed using Unity.

### Conclusions

In conclusion, although there were significant concerns about the public health implications of increased video game playtime during lockdowns, this research has failed to corroborate these concerns. This will be of interest for future pandemic modeling, which may consider potential harms such as disordered gaming.

## Abbreviations

ARMA: autoregressive–moving-average
OxCGRT: Oxford COVID-19 Government Response Tracker
